# From prescription to guidance: a European framework for generic competencies

**DOI:** 10.1007/s10459-019-09910-8

**Published:** 2019-08-26

**Authors:** Jessica E. van der Aa, Anna J. M. Aabakke, Betina Ristorp Andersen, Annette Settnes, Peter Hornnes, Pim W. Teunissen, Angelique J. Goverde, Fedde Scheele

**Affiliations:** 1 Department of Research and Education, OLVG Hospital, Amsterdam, The Netherlands; 2 Athena Institute, Faculty of Science, VU, Amsterdam, The Netherlands; 3 Department of Obstetrics and Gynaecology, Herlev University Hospital, Herlev, Denmark; 4 European Network of Trainees in Obstetrics and Gynaecology (ENTOG), Brussels, Belgium; 5 Department of Gynaecology and Obstetrics, North Zealand Hospital, University of Copenhagen, Copenhagen, Denmark; 6 European Board and College of Obstetrics and Gynaecology (EBCOG), Brussels, Belgium; 7 Department of Obstetrics and Gynaecology, Amsterdam UMC, VU University Medical Centre, Amsterdam, The Netherlands; 8 School of Health Professions Education (SHE), Faculty of Health Medicine and Life Sciences, Maastricht University, Maastricht, The Netherlands; 9 Department of Reproductive Medicine and Gynaecology, University Medical Centre, Utrecht, The Netherlands

**Keywords:** CBME, Change management, Curriculum development, Generic competencies, Implementation, OBGYN, PGME

## Abstract

In postgraduate medical education, required competencies are described in detail in existing competency frameworks. This study proposes an alternative strategy for competency-based medical education design, which is supported by change management theories. We demonstrate the value of allowing room for re-invention and creative adaptation of innovations. This new strategy was explored for the development of a new generic competency framework for a harmonised European curriculum in Obstetrics and Gynaecology. The generic competency framework was developed through action research. Data were collected by four European stakeholder groups (patients, nurses, midwives and hospital boards), using a variety of methods. Subsequently, the data were analysed further in consensus discussions with European specialists and trainees in Obstetrics and Gynaecology. These discussions ensured that the framework provides guidance, is specialty-specific, and that implementation in all European countries could be feasible. The presented generic competency framework identifies four domains: ‘Patient-centred care’, ‘Teamwork’, ‘System-based practice’ and ‘Personal and professional development’. For each of these four domains, guiding competencies were defined. The new generic competency framework is supported by European specialists and trainees in Obstetrics and Gynaecology, as well as by their European stakeholders. According to change management theories, it seems vital to allow room for re-invention and creative adaptation of the competency framework by medical professionals. Therefore, the generic competency framework offers guidance rather than prescription. The presented strategy for competency framework development offers leads for implementation of competency-based medical education as well as for development of innovations in postgraduate medical education in general.

## Introduction

Patients expect their physicians to not only be experts in their medical field, but also to work effectively in teams, deliver patient-centred care and communicate well with patients and colleagues for the sake of quality of care and patient safety (Gillam and Siriwardena 2014; Goleman 2006). In medical education, these non-technical skills, the underlying knowledge and the professional attitudes through which they are displayed, are regarded as generic competencies (Frank et al. 2015; Swing 2007). The acquisition and evaluation of generic competencies are implemented in medical education by embedding them in competency frameworks (Harden et al. 1999). Leading examples of internationally applicable competency frameworks that have been developed through extensive research are the ‘Canadian Medical Education Directions for Specialists (CanMEDS)’, the ‘Core Competencies’, the ‘Tomorrow’s Doctor’ and the ‘Scottish Doctor’ (Frank et al. 2015; Rubin and Franchi-Christopher 2002; Simpson et al. 2002; Swing 2007). These frameworks describe the required competencies into detail, based on the conviction that offering detail in learning and assessment outcomes facilitates implementation of the frameworks (Bank et al. 2017; Hawkins et al. 2015).

However, recent research shows that the implementation of generic competency frameworks in postgraduate medical education (PGME) remains challenging (Hawkins et al. 2015; ten Cate and Scheele 2007). For instance, medical specialists have difficulties in applying the competency frameworks to their formal entrustment decisions on independent performance of trainees (van Loon et al. 2016). Despite the high level of detail in most competency frameworks, resistance to implementation of competency-based medical education (CBME) is reported in the literature (Hawkins et al. 2015). The resistance is, for instance, attributed to the administrative challenges that implementation of CBME brings as well as to inconsistencies in language and definitions in CBME (Boyd et al. 2018; Hawkins et al. 2015; Ringsted et al. 2006; Ten Cate 2017). However, we suspect that this resistance is directed to the concept of CBME, rather than to the reported executional challenges. To gain a better understanding of the resistance to implementation of CBME, and hence of generic competency frameworks, we felt the need to take a step back and explore whether alternative strategies in CBME design may enhance implementation of CBME in PGME.

We built our change management perspective by first reviewing relevant management literature and subsequently formulating potential requirements for CBME design, to generate an understanding of how this design can be better targeted at successful implementation. In our exploration, we made use of management and implementation literature that provides theories and practices in general (Klein and Sorra 1996), and for healthcare organisations in particular (Andreasson et al. 2018; Ferlie et al. 2005; Mintzberg 1983). This body of work offers valuable considerations for the design, development and implementation of innovations in PGME.

To start with, well-established organisational management theories of Mintzberg (1983) identify hospitals as organizations of ‘professional bureaucracy’, in which work and decision-making is decentralised to provide the required autonomy for professionals, in this case the medical professionals (Mintzberg 1983, 2012). This is a valuable theory as we continue to explore the role of medical professionals. A study by Andreasson et al. (2018) found that managers in healthcare often demonstrated a ‘distanced coaching/dumping style’ by leaving all responsibility for the implementation of an innovation to medical professionals (Andreasson et al. 2018). As a result, medical professionals resorted to ‘decoupling’ of the innovation (Meyer and Rowan 1977), meaning that change was implemented in the organisational structure, while daily routines and practices remained the same (Paradis 2017). Through the process of decoupling, medical professionals could leave those activities undisturbed that they considered most important.

In a similar vein, Numerato et al. (2012) concluded that in the processes of policy implementation and management, medical professionals may negotiate and reinterpret standardised management rules to maintain their clinical autonomy (Numerato et al. 2012). As Radaelli et al. (2017) explained, in hospitals, the medical professionals’ position as knowledge experts ensures that they have autonomy in choosing whether and how to adopt a radical innovation (Radaelli et al. 2017). Hence, successful initiation of innovation in hospitals depends heavily on involving the expert knowledge of medical professionals (Radaelli et al. 2014).

All these studies showed that medical professionals ‘re-invent’ innovations before and during implementation processes. The concept of ‘re-invention’ was described by Rogers (2010), who explained it as ‘the degree to which an innovation is changed or modified by a user in the process of its adoption and implementation’, which should not be seen as a distortion of the original concept of the innovation (Rogers 2010). Thus medical professionals need room for re-invention to feel a sense of ownership and to implement an innovation. These findings are in line with the self-determination theory, which stresses the importance of feelings of ownership and autonomy in professionals to enhance their intrinsic motivation to perform their jobs well (Deci et al. 2017).

The importance of a sense of ownership in medical professionals in the implementation of changes is also underlined by literature regarding change management in PGME. Medical professionals play a crucial role in the adaptation of PGME innovations to the existing routines of the workplace, which again enhances ownership (Bank et al. 2015; Fokkema et al. 2013). Besides, as Koksma and Kremer (2019) suggest, quality performance requires room for creativity and learning, and both creativity and learning are only inhibited by objectives that are defined too rigidly (Koksma and Kremer 2019). Hence to enhance the implementation of CBME in PGME, the literatures suggests that there is a need for CBME design that allows for re-invention and creative adaptation by medical professionals. This need may be fulfilled by developing generic competency frameworks that provide guidance, rather than detailed prescriptions of competencies. After all, if medical professionals experience increased ownership of the framework, their autonomous and intrinsic motivation to implement the framework is enhanced, which further stimulates the implementation of the framework.

Recently, the European Board and College of Obstetrics and Gynaecology (EBCOG) initiated the development of a harmonised European curriculum in Obstetrics and Gynaecology (OBGYN) through the Project for Achieving Consensus in Training (PACT) (van der Aa et al. 2016). European harmonisation of PGME aims to improve the quality of training, enhance the international exchange of best practices and increase the mobility of trainees and medical specialists in Europe (van der Aa et al. 2016). Harmonisation is regarded as the establishment of common standards in education, while maintaining institutional autonomy rather than creating uniformity (Patricio and Harden 2010).

This objective of European harmonisation aligns with the suggested strategy for CBME design and leaves room for medical professionals to re-invent and creatively adapt the competency frameworks (Karle et al. 2008). This is of particular importance in Europe, where contexts are highly variable while the implementation of the harmonised PGME curriculum should be feasible in each country (Prideaux 2018). Therefore, we chose the strategy of developing a generic competency framework for guidance, rather than adopting one of the existing competency frameworks (Frank et al. 2015; Simpson et al. 2002; Swing 2007) as the harmonised European OBGYN curriculum. To ensure the integration of competencies that are OBGYN-specific and that meet society’s needs, the newly developed framework takes into account the perspectives of different stakeholders, including physicians, nurses and patients (Frank et al. 2015; Green et al. 2009; Neufeld et al. 1998; Prideaux 2018; Swing 2007; van der Lee et al. 2013).

This article describes the strategy and the process of developing a generic competency framework that facilitates a harmonised European OBGYN curriculum while allowing room for re-invention and creative adaptation. We aimed to answer the following research question: Based on a change management perspective, which generic competencies do European OBGYN specialists need to develop during training, according to the stakeholders? In order to optimally involve the stakeholders in this research, we chose an action research approach.

## Methods

The research process was initiated and led by two researchers in medical education who both have a special interest in OBGYN (JEA and FS). The entire research team consisted of the two primary researchers, four European OBGYN specialists, one European OBGYN trainee and four stakeholder representatives.

In a face-to-face strategy meeting, the main principles of the research were determined. Based on a change management perspective, the research aimed to deliver a generic competency framework for European OBGYN specialists that (a) offers guidance rather than prescription, (b) for which implementation is feasible in all European countries, and (c) is OBGYN specific. It was therefore decided to develop a framework based on CBME, but not to adopt any of the existing competency frameworks.

### Action research objectives

As we aimed to produce knowledge and experiences for PGME in general, action research was chosen as a method to involve the target audience of the generic competencies of OBGYN specialists in Europe as stakeholders in the research. Although action research is scarcely used in research and development of healthcare education, it is valued for its integration of academic and practical knowledge (Delany and Golding 2014; Gleeson 2010; Meyer 2000; Vaughan et al. 2012). It allows different stakeholders to actively participate in the entire research process, for instance in finding scientific answers to research questions, exploring practical implications of the research, and generating new knowledge regarding the issue under investigation (Meyer 2000).

We aimed to foster a collaborative culture with a collective approach by discussing all steps in the research process within the entire research team (Lingard et al. 2008). Finally, we aimed to generate knowledge for all stakeholders that was established through teamwork, to stress the shared responsibility and shared gains of improving generic competencies of European OBGYN specialists.

### Stakeholder representatives and participants

To ensure the development of an OBGYN-specific framework, the research also involved professionals and consumer groups in society who are directly affected by European OBGYN specialists and by the generic competencies that they do or do not display. Stakeholders were identified through stakeholder mapping, which means that all potential stakeholders were distinguished and prioritized according to the focus of the research. As a result, four stakeholder groups were identified: (1) European patients who underwent gynaecological and obstetrical care, and three groups of professionals who collaborate closely with European OBGYN specialists, being (2) nurses, (3) midwives and (4) hospital board members.

For all four identified stakeholder groups, representative organisations were selected and invited to participate in the research. This selection relied, for instance, on experiences in previous national or international collaborative projects. Patients were represented by a foundation that stands for the rights of women undergoing obstetrical care. The organisation expanded their target audience to also include women undergoing gynaecological care. Nurses were represented by a training institute for obstetrical nurses, which expanded its target audience to also include gynaecological nurses. Midwives were represented by a training institute for midwives in primary and secondary care. Hospital board members were represented by the board of a large teaching hospital.

To ensure close collaboration between the four stakeholder representatives and to allow for frequent group discussions, all representatives were deliberately chosen from within the Netherlands. All four stakeholder groups identified potential participants and appealed to relevant organisations in all European countries for data collection in order to ensure a wide representation of European patients, nurses, midwives and hospital board members. Although each stakeholder representative designed their own approach to the research, they frequently exchanged information on appropriate methods and on progress.

### Stages of the research

**Fig. 1:**
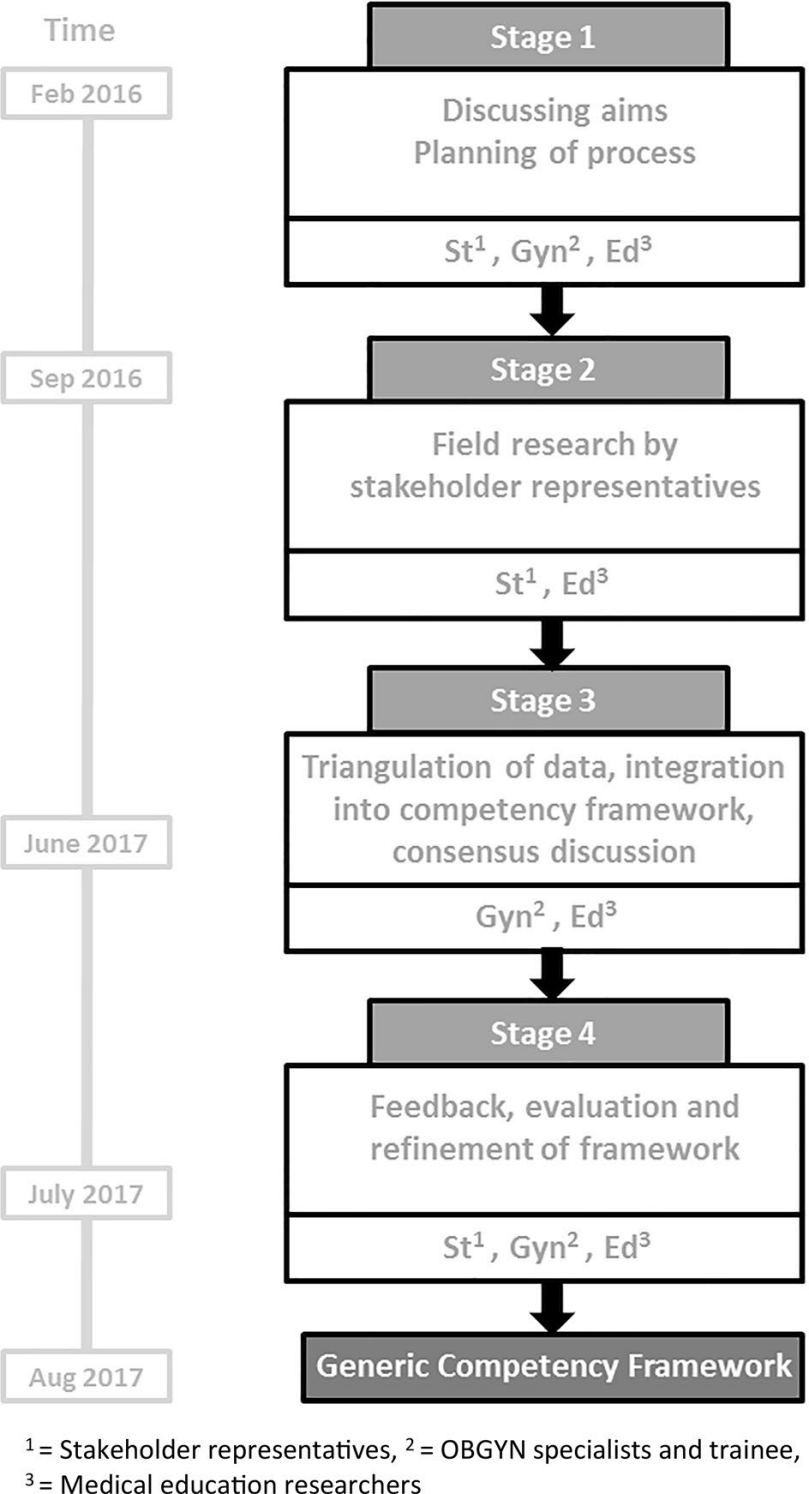
Research stages of the development of a generic competency framework for European OBGYN specialists. ^1^Stakeholder representatives, ^2^OBGYN specialists and trainee, ^3^Medical education researchers

In general, action research includes different stages with different purposes, such as planning, development or action, reflection, modification and consensus formation. In this study, we identified four stages in the process, which are illustrated in Fig. 1.

After having discussed the research aims and having planned the process with the entire research team (stage 1), all four stakeholder representatives explored answers to the research question on behalf of the group they represented. The stakeholder representatives determined their own methods to collect data and select participants. They did so by considering the convenience of participant selection methods, the usefulness of platforms to recruit participants, the appropriateness of different research methods and the different contexts to collect data. The stakeholders collected data through reviews of literature (nurses), consensus meetings (nurses and patients), questionnaires (patients and midwives) and semi-structured interviews (hospital board members) (Meyer 2000). Ethical approval was waived for various methods of data collection that included participants (OLVG hospital, Amsterdam, the Netherlands, case WO 16.593, Ethical Review Board of the Netherlands Association for Medical Education file number 787).

During stage 2 of the research, the stakeholder representatives collected and analysed their data and held regular meetings to discuss the progress and methods of their research approaches. The principal researcher (JEA) was present at all these meetings to offer advice on as well as to guarantee quality of the research process. During this stage, the entire research team gathered in a face-to-face meeting to discuss progress and preliminary results and to evaluate the process, as well as to, validate the data. Eventually, all stakeholder representatives presented their data in written reports. Altogether, fifteen European nationalities were represented in the participant groups of the research performed by the stakeholder representatives.

In stage 3 of the research, the OBGYN specialists integrated all the data, taking into account the strategic principles of the research. They extracted key phrases and key elements of the reports and categorized them into domains. Subsequently, the two main researchers (JEA and FS) and the OBGYN trainee joined the consensus discussion to discuss the process so far, to further triangulate the data and to develop clarity in the formulation of the different domains that emerged. In the triangulation of data, the strategic principles of ensuring the feasibility of implementation and ensuring the guiding nature of the competency framework were leading. For each domain, the researchers formulated underlying descriptions of competencies. These descriptions determined the level of detail of the generic competency framework. Areas of uncertainty were identified and discussed until consensus was reached about clarification. This resulted in a preliminary generic competency framework.

Through the final feedback round (stage 4), the stakeholder representatives reflected on how the data were integrated in the preliminary competency framework. The framework was then modified according the feedback, resulting in the final format of the generic competency framework. Subsequently, the framework was presented to members of the council of EBCOG and of the European Network of Trainees (ENTOG), who represent OBGYN specialists and trainees from 36 European countries. They evaluated the framework’s design and the detail with which the competencies were described in relation to the feasibility of its implementation in all European countries.

## Results

Table 1 summarises all topics of competencies that were described as relevant in the stakeholder reports. There was a considerable overlap in these topics, between the reports. For instance, all stakeholders identified the topics of providing personalised or individualised or patient-centred care and of communicating clearly and empathetically as vital.

**Table 1:** Summary of results of research by stakeholder representatives

Input from patients	Input from nurses	Input from midwives	Input from hospital boards
Basic connection skills: make real contact with the person	Pursue empathic patient-centred care	Communicating with the patient	Effective and empathetic communication
Give individualized care	Master patient safety concepts and skills	Relationship and attitude of midwives and gynaecologist	Diversity knowledge
Informed choice: give information, choices, and inform about the woman’s rights	Show collaborative practice as an overarching mastery	Influences at the organizational level	Adaptability to diversity
Pay attention to setting and context	Understand the nurse’s position in transition from a subordinate role to a vital and equally valuable professional role	Personal abilities of the gynaecologist	Solid foundation of medical expertise
	Be aware of the potential beneficial and detrimental effects of power within health care teams	Importance of education	Ensuring and improving quality of care
	Understand the concepts of good leadership		Co-creation
			Healthcare progression

**Fig. 2:**
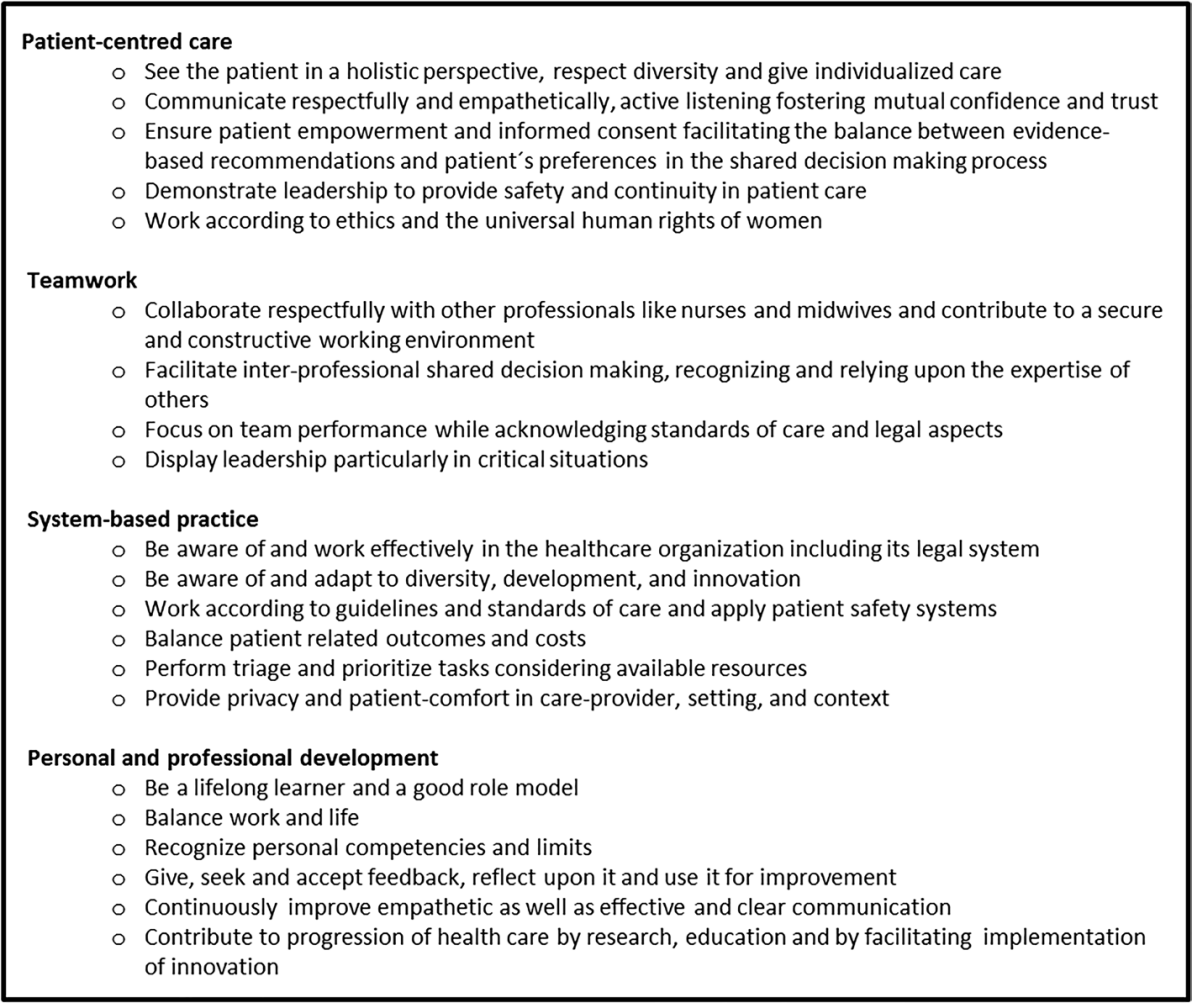
Guiding generic competency framework for European OBGYN specialists in four domains

The OBGYN specialists, the trainee and the two main researchers designed the framework based on input from the stakeholder reports. In the design, they considered the desired guiding nature of the framework, the required feasibility of implementation across Europe, and the specialty-specific nature. In line with these principles, they considered the relevance and applicability of the topics that were described in the reports. Figure 2 shows the final generic competency framework for the European OBGYN specialist, which describes competencies in four domains: ‘Patient-centred care’, ‘Teamwork’, ‘System-based practice’ and ‘Personal and professional development’.

Comparison of the domains and competencies of the framework with the topics in the stakeholder reports shows how the framework was designed to align with its intended guiding nature. For instance, the patients’ representatives thoroughly described how an OBGYN specialist should communicate with patients, make them comfortable and inform them, but also offer them choices. These requirements were described in detail. As the researchers considered that it would not be feasible to adopt these specific requirements in all European countries, they were described as competencies that invite OBGYN specialists to set conditions for shared decision making with each patient. Therefore, the competencies do not prescribe what OBGYN specialists must or must not do, but allow medical professionals to adapt them to their context.

As a second example, the nurses as well as the midwives and hospital board members stressed the importance of collaboration competencies for OBGYN specialists. These requirements were integrated into the framework in the domain of ‘Teamwork’. The competencies were described from a general perspective, to allow room for re-invention and creative adaptation in the workplace, thus ensuring feasibility of implementation in different European contexts. As the reports also showed that collaborative practices in different European contexts are affected by relations, hierarchy and other cultural factors, OBGYN specialists and trainees can follow the guiding principles of the domain of ‘Teamwork’ without disturbing local customs and practices. In this way they are encouraged to establish teamwork that fits the local contexts, and hence they may be more willing to develop and display the related competencies.

Some requirements that were described in the stakeholder reports (Table 1) were not integrated in the final framework (Fig. 2), because they did not align with the strategic principles of the framework development, as was evaluated in stage 3 of the research. As an example, midwives concluded from their data that OBGYN trainees should follow a compulsory internship in midwifery-led care to become familiarised with midwifery-care perspectives of pregnancy, labour and birth. In stage 3 of the research, it was considered that this requirement is quite prescriptive, as it suggests that the required competencies can only be acquired through a specific internship. Also, it may not be feasible in many European countries to organise midwifery-led care internships, due to great variation in organisation of care, collaboration with midwives, etcetera. Therefore, this stakeholder’s requirement was considered too prescriptive and to leave too little room for re-invention and creative adaptation and hence it was not included in the generic competency framework. This example illustrates the importance of stage 3 in the research process to generate a competency framework that answers to the stakeholders’ needs, yet is feasible for varying contexts.

When this framework was presented to the Council Meeting of EBCOG, all the representatives of the European countries’ national societies and trainee organisations agreed that the level of detail of the competency framework complied with the aims of the harmonised curriculum as well as with their needs for guidance to train and to assess generic competencies.

## Discussion

### Summary of findings and contribution to the current literature

This research reports on how a generic competency framework for a harmonised European OBGYN curriculum was designed as a guiding framework. Generic competencies that European OBGYN specialists need to attain during PGME are described in four domains: ‘Patient-centred care’, ‘Teamwork’, ‘System-based practice’ and ‘Personal and professional development’. The strategy was to develop a competency framework that provides guidance, is specialty-specific, and for which implementation is feasible in all European countries.

Regarding the content of the European generic competency framework, our research resulted in a competency framework in which competencies related to personal and professional development of the medical specialist were considered essential. Compared to existing competency frameworks, the personal aspects of the medical specialist and the need to develop competencies at this level take a more prominent position in the European generic competency framework (Frank et al. 2015; Rubin and Franchi-Christopher 2002; Simpson et al. 2002; Swing 2007). Regarding the strategy of the European generic competency framework, our research resulted in valuable new perspectives. With specific strategic principles in mind, a generic competency framework was developed that allows for re-invention and creative adaptation by medical professionals, which, according to change management literature, will support the implementation of the framework.

This study describes a first step in the exploration of an alternative strategy for CBME design, namely to consider the implementation of CBME from a change management perspective. This is necessary, because the introduction of CBME has caused a major paradigm shift in medical education and was often criticised in the literature (Ringsted et al. 2006; Ten Cate 2017; ten Cate and Scheele 2007; Whitehead et al. 2013; Whitehead and Kuper 2015). Evaluation of CBME has yielded criticism regarding its administrative burdens, its inconsistencies in language and approach, and its varying content (Hawkins et al. 2015). Unfortunately, the academic discourse offers rather limiting replies to these criticisms (Boyd et al. 2018). In an attempt to move the discussion forward, we used these criticisms as opportunities to apply a new strategy for CBME design and to explore constructive ways to improve the implementation of CBME. Our research shows that an alternative approach to CBME development resulted in a widely recognized generic competency framework that allows room for re-invention and adaptation of the required competencies. European OBGYN specialists who aim to harmonise PGME indicated that they feel positive about this strategy and that they support the proposed guiding principles.

The struggle against the increasing rigidity and the predominance of prescriptive tools is similarly recognised in other major themes in medical education, such as quality (Koksma and Kremer 2019), reflection (de la Croix and Veen 2018), communication (Mole et al. 2016) and accreditation (Akdemir et al. 2017). In these areas as well, pleas are made to embrace diversity, reduce the number of standards, set fuzzy goals, and allow room for professional creativity and learning in the quest for quality improvement (Akdemir et al. 2017; de la Croix and Veen 2018; Koksma and Kremer 2019; Mole et al. 2016).

Therefore, we call for PGME design that is based on guiding principles, rather than on prescribing standards, to allow for re-invention and creative adaptation of CBME.

### Practical implications

This research offers an alternative approach for CBME development from a novel perspective, which may serve as an opportunity to rethink CBME design. This may be valuable for medical education in general, and for the harmonisation of PGME in particular.

Assuming that the generic competency framework for the European harmonised OBGYN curriculum will be adopted throughout Europe, questions arise on how to ensure the implementation and, subsequently, the quality control of the framework (Dauphinee et al. 2018). We suggest that local re-invention and creative adaptation of the framework will stimulate the development of local frameworks by local medical professionals. These local frameworks should be more useful for medical professionals to implement in their own workplace, by being transparent for all concerned, possibly more detailed and more in line with the needs of patients and healthcare providers within local contexts. This process could, for instance, be guided by communities of learners who operate at local levels. As Koksma and Kremer (2019) described, we suggest to aim for ‘cultivating a learning culture to bring quality improvement into a learning era’ (Koksma and Kremer 2019).

However, the practical application of the outcomes of our study does depend on the availability of opportunities for local re-invention and creative adaptation to local contexts. This may not be feasible or cannot be expected in all contexts and from all medical professionals in Europe. Some may need more detailed and prescriptive methods. In these situations, existing competency frameworks offer valuable handbooks, since they provide more detailed descriptions of competencies that are very much in line with the framework that was developed (Frank et al. 2015; Rubin and Franchi-Christopher 2002; Simpson et al. 2002; Swing 2007).

### Strengths, limitations and implications for future research

This research provides a novel perspective on the design and implementation of CBME through integration of management literature concepts and medical education research. A strength of the study is the involvement of stakeholders from all over Europe, which ensured representation of many different European contexts in the study. A limitation of the study is that the representation of stakeholders from some European countries was limited. This was caused by the experience of great variation between different European countries on whether they valued the involvement of stakeholders, and whether the stakeholders experienced support for their participation in the research. For instance, the midwives experienced very different involvement between different countries, and the nurses did not experience any support from their international professional platforms within Europe.

Future research should evaluate whether the presented strategy will actually have the anticipated and desired effects.

## Conclusions

This study aimed to deliver a generic competency framework for a harmonised European OBGYN curriculum. To enhance the implementation of CBME in PGME, we explored an alternative strategy in CBME design from a change management perspective. We have demonstrated that a generic competency framework can be developed that is supported by OBGYN specialists, OBGYN trainees and stakeholders of the OBGYN specialist from the entire Europe. This was achieved by involving all three groups throughout the entire process of research and development. It was key to allow room for re-invention and creative adaptation of the competency framework by medical professionals. This was ensured by designing a generic competency framework that offers guidance rather than prescription. The presented strategy of competency framework development offers leads for effective implementation of CBME, which may eventually benefit quality of care and patient safety.
